# Impact of body tilt on the central aortic pressure pulse

**DOI:** 10.14814/phy2.12360

**Published:** 2015-04-12

**Authors:** Corina Rotaru, Lucas Liaudet, Bernard Waeber, François Feihl

**Affiliations:** 1 Division de Physiopathologie clinique, Centre Hospitalier Universitaire Vaudois, University of LausanneLausanne, Switzerland; 2 Service de Médecine intensive adulte, Centre Hospitalier Universitaire Vaudois, University of LausanneLausanne, Switzerland

**Keywords:** Aorta, blood pressure, diastole, hemodynamics, posture, pulse wave analysis

## Abstract

The present work was undertaken to investigate, in young healthy volunteers, the relationships between the forward propagation times of arterial pressure waves and the timing of reflected waves observable on the aortic pulse, in the course of rapid changes in body position. 20 young healthy subjects, 10 men, and 10 women, were examined on a tilt table at two different tilt angles, −10° (Head-down) and + 45° (Head-up). In each position, carotid-femoral (*T*_cf_) and carotid-tibial forward propagation times (*T*_ct_) were measured with the Complior device. In each position also, the central aortic pressure pulse was recorded with radial tonometry, using the SphygmoCor device and a generalized transfer function, so as to evaluate the timing of reflected waves reaching the aorta in systole (onset of systolic reflected wave, sT1r) and diastole (mean transit time of diastolic reflected wave, dMTT). The position shift from Head-up to Head-down caused a massive increase in both *T*_ct_ (women from 130 ± 10 to 185 ± 18 msec *P* < 0.001, men from 136 ± 9 to 204 ± 18 msec *P* < 0.001) and dMTT (women from 364 ± 35 to 499 ± 33 msec *P* < 0.001, men from 406 ± 22 to 553 ± 21 msec *P* < 0.001). Mixed model regression showed that the changes in *T*_ct_ and dMTT observed between Head-up and Head-down were tightly coupled (regression coefficient 2.1, 95% confidence interval 1.9–2.3, *P* < 0.001). These results strongly suggest that the diastolic waves observed on central aortic pulses reconstructed from radial tonometric correspond at least in part to reflections generated in the lower limbs.

## Introduction

Physiologists have had a very long-standing interest in the mechanisms governing the shape of the arterial pressure pulse. More than a century ago, Otto Frank introduced his classical mathematical approach known as the Windkessel model, whose heuristic value is still recognized today (Westerhof et al. 2009). In his original paper (Frank 1899), Frank attempted to explain the deviations observed between model predictions and experimental observations on account of nonlinear pressure–volume behavior of large arteries and/or time-varying peripheral resistance. Later on, however (Frank 1905), he linked these deviations to the finite propagation velocity of pressure waves along arteries in the forward (from heart to periphery) and backward directions (reflections from the periphery toward the heart), never quite resolving the inherent contradiction of a lumped model (Windkessel) that would possess transmission line properties (Parker 2009; Nichols et al. 2011a). It was for the next generations of physiologists (Dow and Hamilton 1939; McDonald 1955; Taylor 1957; Wormersley 1958; O'Rourke 1967; Nichols et al. 1977) to solve this issue, providing a consistent framework for explaining the oscillatory behavior of the arterial system, both in the time and frequency domain, across age groups, genders, health conditions, and species (reviewed in Nichols et al. 2011a; O'Rourke and Yaginuma 1984; Parker 2009).

At any point in the arterial system, and in particular in the ascending aorta, the observed pressure pulse results from the summation of forward and backward (reflected) pressure waves. Depending on conditions, reflected waves may reach the ascending aorta in systole, diastole, or both. An essential determinant in that respect is the propagation velocity of pressure waves in either direction along the arterial tree (pulse wave velocity, PWV). Due to the Moens–Korteweg equation, stiff arteries favor a high PWV, thus short propagation times to and from reflection sites, thus arrival of reflected waves in the ascending aorta before the end of ventricular ejection, thus systolic augmentation of aortic pressure (O'Rourke and Hashimoto 2007). By the same token, compliant arteries favor the augmentation of aortic pressure by reflected waves during diastole. Diastolic augmentation may also result from multiple, back, and forth reflections and rereflections between aorta and periphery (Nichols et al. 2011c). Finally, due to the nonlinear stress–strain relationship characteristic of arterial walls (Nichols et al. 2011b), PWV in any given artery is directly related to its transmural pressure. When the latter is low, therefore (as occurs in shock or during a Valsalva maneuver), reflected waves reach the aorta predominantly in diastole (Nichols et al. 2011d).

Many factors may affect the shape of the aortic pressure pulse in daily life. Prominent among them should be changes in body position, which trigger reflex changes in stroke volume and peripheral resistance, potentially affecting the amplitude of both the forward and the reflected pressure waves. In addition, PWV, and therefore wave propagation times, are also subject to gravitational effects via changes in the transmural pressure of arteries, due to the fact that hydrostatic forces are integrally transmitted within (Katkov and Chestukhin 1980) but not outside the vessel lumen (Meyer et al. 2002). In spite of this rationale, the effects of body position on the central aortic pulse have not been investigated in detail. Some studies have described the changes in aortic pulse associated with passive leg elevation (Kamran et al. 2009; Heffernan et al. 2010), tilt (Kroeker and Wood 1955; Huijben et al. 2012), or switching from supine to sitting (Jaccoud et al. 2012; Heim et al. 2013). All provide information on systolic, but only two (Jaccoud et al. 2012; Heim et al. 2013) on diastolic events. Only in the study by Huijben et al. (2012) was the recording of aortic pulse associated in each tested position with concomitant measurements of PWV.

The present work was designed to overcome these limitations. Young healthy subjects of both genders were investigated on a tilt table. We hypothesized a close coupling between the tilt-induced changes in: (1) timing of the reflected waves seen in both systole and diastole on the central aortic pulse and (2) forward propagation times of pressure waves. The latter were estimated, not only in trunk vessels as done in the aforementioned study by Huijben et al. (2012) but also in leg arteries, whose transmural pressure is likely to vary to a greater extent with body position.

## Methods

### Subjects

Twenty healthy Caucasian subjects, 10 men and 10 women, aged from 18 to 40 years, without any medication, were recruited by advertisement. Exclusion criteria were: overweight or obesity (BMI > 25 kg/m²), sedentary lifestyle as screened by a questionnaire (Mader et al. 2006), history of orthostatic hypotension, any kind of medication, and nicotine addiction. All participants gave free and informed consent in written. The study protocol was approved by the local Ethical Committee.

### Pulse wave analysis

The pulse waveform in the right radial artery was recorded with applanation tonometry using the SphygmoCor device (Atcor Medical, Sydney, Australia), as previously described by our group (Delachaux et al. 2006; Gojanovic et al. 2009; Dischl et al. 2011; Jaccoud et al. 2012; Heim et al. 2013). Recordings meeting the quality criteria proposed by the manufacturer were obtained in triplicate. Using the generalized transfer function approach, as implemented in the SphygmoCor software, the radial recording was transformed into a central pulse waveform. The waveforms were calibrated using oscillometric blood pressure measured on the same arm immediately before tonometry.

All subjects were in sinus rhythm during the experiment. Recordings containing ectopic beats were discarded.

### Pulse pressure forward propagation times and pulse wave velocity

The time intervals separating the carotid from the femoral (*T*_cf_), the femoral from the tibial (*T*_ft_), and the carotid from the tibial arterial pulse (*T*_ct_) were obtained from recordings carried out simultaneously on these three arteries, using the the Complior device and version 1.3.0j of the Complior SP software (Alam Medical, Vincennes, France) (Laurent et al. 2006). All recordings were carried out in triplicate. From the distances between the recording sites, measured with a ruler, the corresponding pulse wave velocities (PWV_cf_ carotid-to-femoral, PWV_ft_ femoral-to-tibial, PWV_ct_ carotid-to-tibial) were calculated.

### Protocol

Each participant was studied only once, between 16:00 and 18:00 h, in a quiet room.

The subject was positioned in the supine position on a tilt table. A pressure cuff was placed on the right arm for the oscillometric measurement of brachial blood pressure (HEM-907-E, Omron Healthcare Europe, Hoofddorp, Netherlands). In order to monitor hemodynamic stability, photoplethysmographic cuff was fitted to the third finger of the left hand, for the continuous monitoring of the blood pressure waveform in the digital arteries, using the Finapres system. The specific apparatus used was the Finometer® PRO (Finapres Medical Systems, Amsterdam, Netherlands), together with version 1.1 of the BeatScope software, which calculates stroke volume (SV) from the analysis of the digital arterial pulse based on the Modelflow method (Wesseling et al. 1993).

Examinations were carried out with the subject in the Head-down and in the Head-up positions, applied in random order. These positions were obtained by setting the tilt table inclination at either −10° (Head-down) or +45° (Head-up) with respect to horizontal. These inclinations were chosen because the tilt table at our disposal did not allow more negative angles, and because a more vertical Head-up position entailed too great a risk of triggering vasovagal reflexes, especially in these young subjects. In each position, and after an initial stabilization period of 5 min, two sets of three radial tonometric recordings (M1 and M2) were taken with the SphygmoCor device. In between M1 and M2, the different forward propagation times and pulse wave velocities were obtained in triplicate with the Complior device, as described above. All measurements were performed by the same investigator. The subjects kept both arms and hands quietly laid on the tilt table, alongside their body, during the whole experiment.

### Analysis of the central pulse waveform

**Figure 1:**
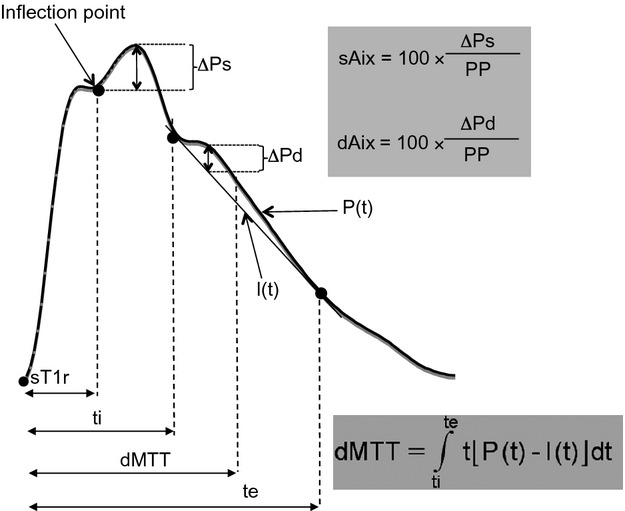
Outline of calculations made on the central aortic pressure waveform. sT1r time of onset of systolic reflected wave, whose arrival in the aorta is denoted by the inflection point. ΔPs difference between peak pressure and pressure at the inflection point. PP pulse pressure. sAix systolic augmentation index. The “diastolic wave” designates the upward convexity visible on the diastolic part of the pressure waveform. Its time of onset (ti) and end (te) are determined by the contact points of the line l(t) in tangent contact with the diastolic profile as shown in Figure1. The algorithm for choosing ti and te is detailed in the Appendix. ΔPd is the maximal vertical distance between the true pressure profile P(t) and l(t). dAix diastolic augmentation index. dMTT mean transit time of the diastolic wave, calculated according to the formula shown, with the origin of time set at the onset of the systolic upstroke.

This analysis has been described in detail in our previous publications (Jaccoud et al. 2012; Heim et al. 2013). A sketched in Figure1, the following parameters were calculated: time of onset of reflected waves reaching the aorta in systole (sT1r), systolic augmentation index (sAix), mean transit time of diastolic wave (dMTT), and diastolic augmentation index (dAix). sT1r and sAix were provided by the Sphysgmocor software, which also calculates a value of sAix corrected for heart rate (sAix@75). dMTT and dAix were obtained from custom software, as described (Jaccoud et al. 2012; Heim et al. 2013). The algorithm used by this software in order to delineate the diastolic wave is detailed in the Appendix.

### Statistical analysis

The variables of interest were compared using analysis of variance for repeated measures. The fixed effects in the model were gender, position, their interaction, and measurement set (M1 or M2) if appropriate. The subject nested with gender was included as a random effect. When the relevant *F* test was significant, further pairwise comparisons were made using modified *t*-tests. The alpha level of all tests was 0.05. Calculations were with the JMP software (version 5, SAS institute, Cary, NC). In addition, the relationships between forward propagation times and timing indices derived from the central aortic waveform were explored with mixed model linear regression, including gender as a fixed covariate, using STATA (version 12; StataCorp LP, College Station, TX). Continuous data are summarized as means and SD.

## Results

The demographic characteristics of the study subjects are shown in Table1. In comparison with women, men were taller, heavier, and slightly older. The two groups were matched for BMI.

**Table 1:** Demographic data.

	Women	Men
Number	10	10
Age (years)	27.9 ± 5.2	24.8 ± 2.3^ns^
Height (cm)	166.2 ± 4.2	180.5 ± 5.3***
Weight (kg)	57.0 ± 5.7	72.1 ± 4.5***
BMI (kg/m²)	21 ± 2	22 ± 2^ns^

^ns^not significant.

^*^^*^^*^P < 0.001 women versus men.

### Basic hemodynamic data

The upper part of Tables2 (women) and 3 (men), shows basic hemodynamic data recorded in duplicate (M1 and M2, before and after the determination of PWV) in Head-up and Head-down position. All of these values were quite similar between M1 and M2, indicating that, in each position subjects were hemodynamically stable.

**Table 2:** Hemodynamic data as a function of body position in women.

Body position	Head up		Head down		*P* values		
Measurement	M1	M2	M1	M2	Body position	M1 vs. M2 Head up	Head down
Heart rate (b/m)	78 ± 11	80 ± 11	64 ± 10	64 ± 10	<0.001	ns	ns
Peripheral BP (mmHg)							
Systolic	112 ± 9	111 ± 9	107 ± 12	108 ± 10	<0.001	ns	ns
Diastolic	74 ± 7	76 ± 7	65 ± 6	66 ± 5	<0.001	ns	ns
Mean	85 ± 7	87 ± 6	79 ± 7	79 ± 6	<0.001	ns	ns
Central BP (mmHg)							
Systolic	96 ± 7	97 ± 7	93 ± 9	93 ± 7	<0.001	ns	ns
Diastolic	75 ± 7	77 ± 6	66 ± 6	67 ± 5	<0.001	ns	ns
Mean	85 ± 7	87 ± 6	79 ± 7	79 ± 6	<0.001	ns	ns
Stroke volume (ml)	49 ± 14	48 ± 14	70 ± 14	70 ± 13	<0.001	ns	ns
Cardiac output (l/min)	3.7 ± 0.8	3.7 ± 0.8	4.4 ± 0.8	4.4 ± 0.9	<0.001	ns	ns

Peripheral BP measured with oscillometry. In each body position, the displayed variables were recorded on two separate occasions (M1 and M2). The measurements of pulse wave velocities and transit times shown in Figure4 were obtained between M1 and M2. BP, blood pressure.

**Table 3:** Hemodynamic data as a function of body position in men.

Body position	Head up		Head down		*P* values		
Measurement	M1	M2	M1	M2	Body position	M1 vs. M2 Head up	Head down
Heart rate (b/m)	76 ± 13	78 ± 9	60 ± 10	60 ± 8	<0.001	ns	ns
Peripheral BP (mmHg)							
Systolic	121 ± 9	121 ± 9	119 ± 7	119 ± 6	<0.001	ns	ns
Diastolic	72 ± 5	75 ± 3	59 ± 6	59 ± 3	<0.001	ns	ns
Mean	85 ± 6	87 ± 5	75 ± 6	76 ± 3	<0.001	ns	ns
Central BP (mmHg)							
Systolic	101 ± 7	102 ± 6	97 ± 5	97 ± 4	<0.001	ns	ns
Diastolic	74 ± 5	76 ± 3	59 ± 5	60 ± 3	<0.001	ns	ns
Mean	85 ± 6	87 ± 5	75 ± 6	76 ± 3	<0.001	ns	ns
Stroke volume (ml)	61 ± 10	63 ± 13	103 ± 16	105 ± 15	<0.001	ns	ns
Cardiac output (l/min)	4.9 ± 1.0	5.0 ± 1.0	6.4 ± 1.4	6.5 ± 1.4	<0.001	ns	ns

Peripheral BP measured with oscillometry. In each body position, the displayed variables were recorded on two separate occasions (M1 and M2). The measurements of pulse wave velocities and transit times shown in Figure4 were obtained between M1 and M2. BP, blood pressure.

As expected, heart rate was substantially higher (by 20%) and stroke volume markedly lower (by 40%), leading to a reduced cardiac output in the Head-up compared to the Head-down position (*P* < 0.001 for both variables). Although statistically significant, the impact of position on peripheral and central systolic blood pressure (BP) was of minor amplitude, whereas, consistent with the change noted in stroke volume, peripheral, and central diastolic BP was clearly higher in the Head-up position. In all conditions, the systolic, but not diastolic BP was higher in the radial artery than in the aorta, so that peripheral pulse pressure was higher than its central counterpart. The peripheral amplification of pulse pressure is a well-known consequence of wave reflection (Nichols et al. 2011d).

Statistically significant gender-related differences in these basic hemodynamic data are displayed in the upper part of Table4. In women compared to men, peripheral but not central systolic BP was lower in both positions, and the switch from Head-down to Head-up induced a less marked rise in peripheral or central diastolic BP in accordance with the expected lower orthostatic tolerance of women (Convertino 1998).

**Table 4:** Statistical comparisons between genders.

Body position	Head up	Head down	Interaction of gender and body position
Heart rate (b/m)	ns	ns	ns
Peripheral BP (mmHg)			
Systolic	0.003	0.003	ns
Diastolic	ns	0.004	0.012
Mean	ns	ns	ns
Central BP (mmHg)			
Systolic	ns	ns	ns
Diastolic	ns	0.004	0.006
Mean	ns	ns	ns
Stroke volume	0.02	<0.001	0.002
Cardiac output	<0.001	<0.001	ns
Wave reflection indices			
Systolic			
sAix (%)	0.004	0.004	ns
sAix@75 (%)	0.005	0.005	ns
sT1r (ms)	ns	0.01	0.031
Diastolic			
dAix (%)	0.006	0.006	ns
dMTT (ms)	<0.001	<0.001	ns
Pulse wave velocity			
Carotid-femoral	ns	ns	ns
Femoral-tibial	ns	ns	ns
Carotid-tibial	ns	ns	ns
Transit times			
Carotid-femoral	ns	ns	ns
Femoral-tibial	<0.001	0.007	ns
Carotid-tibial	ns	0.007	0.073

sAix systolic augmentation index, sAix@75 systolic augmentation adjusted for a heart rate of 75beats/min, sT1r time to onset of systolic reflected wave dAix diastolic augmentation index, dMTT mean transit time of diastolic reflected wave. ns nonsignificant women versus men.

### Central aortic pulse waveforms

**Figure 2:**
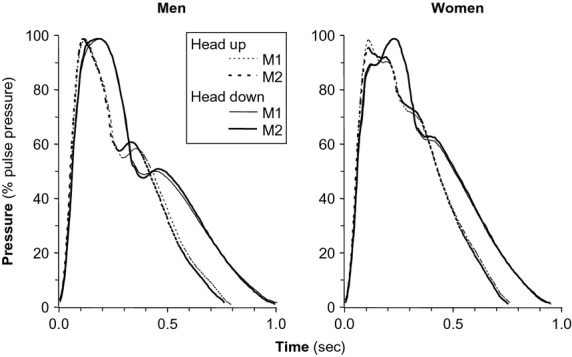
Ensemble-averaged aortic pulse waveforms in women and men in the Head-up and Head-down positions.

Figure2 shows the central aortic pressure waveforms, ensemble averaged for the two stages of the protocol, over the 10 men and over the 10 women enrolled in the study. In both groups, these profiles appear reproducible (compare M1 with M2) and their longer duration in the Head-down position directly reflects the lower heart rate in this condition. In women, the antegrade and reflected systolic waves appear clearly delineated, with the latter augmenting peak systolic pressure in the Head-down position only. The switch from Head-up to Head-down appears to delay the diastolic wave. On the ensemble-averaged profiles of men, the inflection point allowing to distinguish the antegrade from the reflected systolic wave in not clearly recognizable, and position affects the timing of the diastolic wave in the same manner as it does in women.

### Indices of wave reflexion

**Figure 3:**
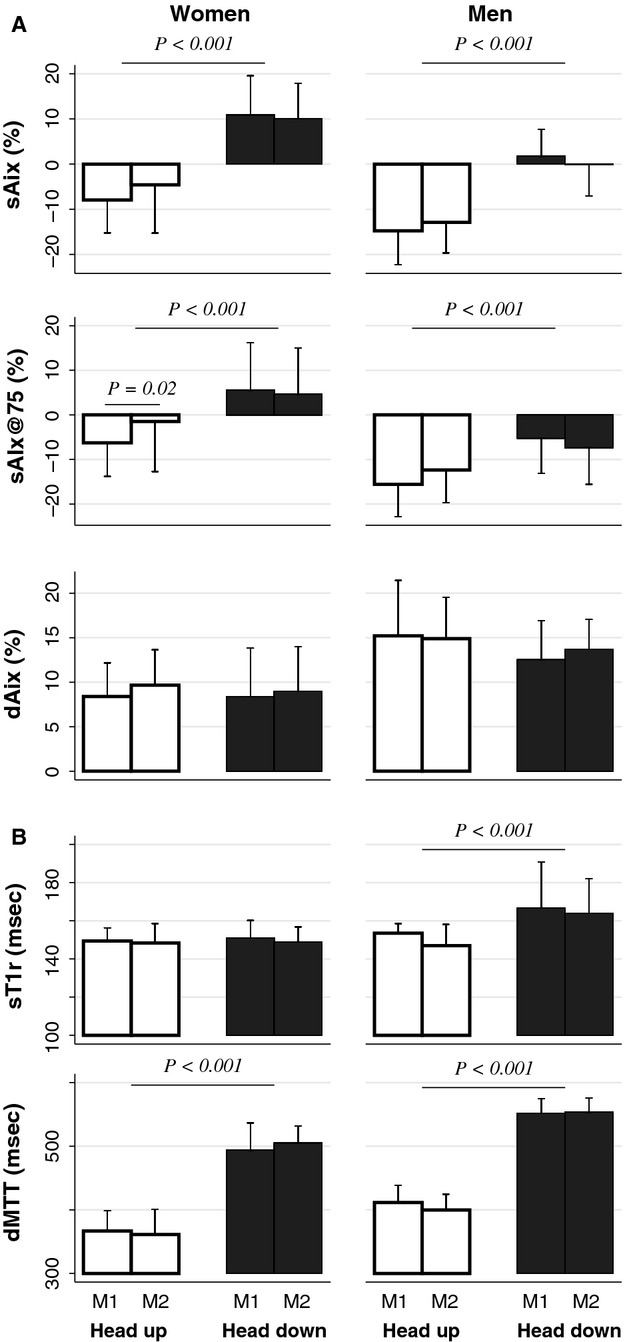
Indices of wave reflection in women and men in the Head-up and Head-down positions. sAix systolic augmentation index. sAix@75 systolic augmentation index corrected for heart rate. dAix diastolic augmentation index. sT1r time to onset of systolic reflected wave, dMTT mean transit time of diastolic reflected wave. In each body position, the displayed variables were recorded on two separate occasions (M1 and M2). The measurements of pulse wave velocities and transit times shown in Figure4 were obtained between M1 and M2.

Results regarding the quantitative indices of wave reflection derived from the central pressure waveforms are shown in Figure3. As expected (Heim et al. 2013), the algebraic values of the systolic augmentation index (whether unadjusted, sAix, or adjusted for heart rate, sAix@75) were higher in women compared to men, whereas the reverse was true for its diastolic counterpart (dAix), and all these gender-related differences were statistically highly significant (Table4, middle part). The impact of body position on these indices was essentially independent of gender, with the shift from Head-up to Head-down increasing sAix as well as sAix@75 and leaving dAix unchanged. Compared to Head-up, Head-down slightly delayed the systolic wave in men only (increase of T1r from Head-up to Head-down, mean ± SD: women +1 ± 8 msec, *P* = 0.74; men +15 ± 16 msec, *P* < 0.001), and markedly delayed the diastolic wave in both genders (increase of dMTT from Head-up to Head-down: women +135 ± 35 msec *P* < 0.001; men +147 ± 31 msec, *P* < 0.001).

### Pulse wave velocity and forward propagation times

**Figure 4:**
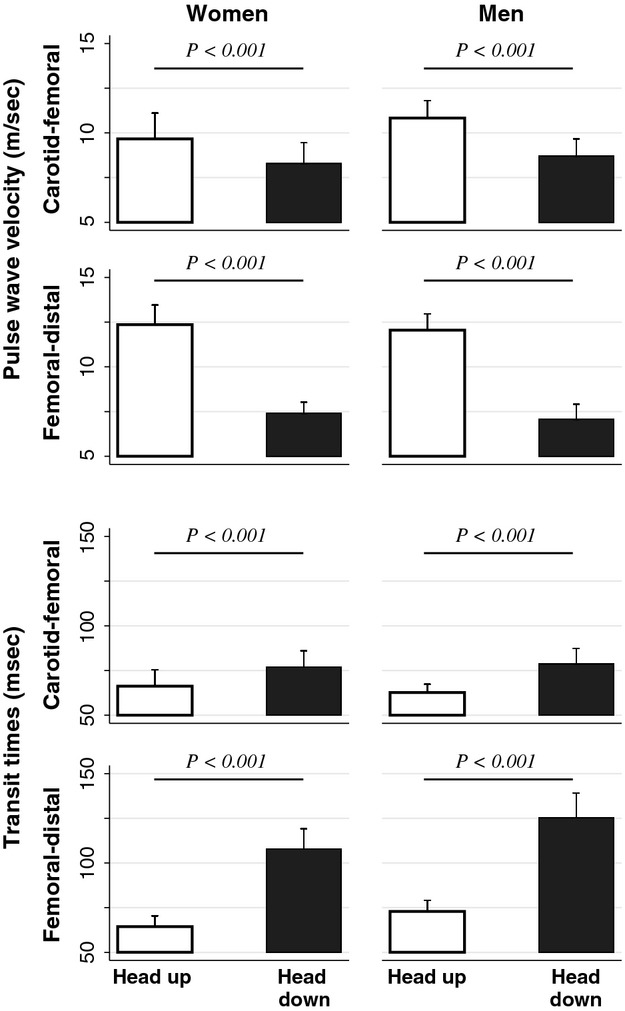
Pulse wave velocities and forward propagation times in women and men in the Head-up and Head-down positions.

As shown in Figure4, the pulse wave velocities measured from the carotid-to-femoral (PWV_cf_), femoral-to-tibial (PWV_ft_), and carotid-to-tibial sites (PWV_ct_) all significantly decreased in both genders from the Head-up to the Head-down position. This effect was highly statistically significant (*P* < 0.001 in all cases) and much more marked for arteries in the lower limb (mean and SD of observed changes in PWV_ft_ from Head-up to Head-down: women −5.0 ± 1.0 m/sec, men −5.0 ± 1.2 m/sec) than for those in the thoracoabdominal compartment (changes in PWV_cf_: women −1.4 ± 1.0 m/sec men −2.1 ± 1.2 m/sec). The various forward propagation times increased correspondingly (mean and SD of observed changes, *P* < 0.001 in all cases: *T*_cf_ women +10.7 ± 7.1; men +16.0 ± 8.4 msec; *T*_ft_ women +43.4 ± 10 msec, men +52.4 ± 16.8 msec).

**Figure 5:**
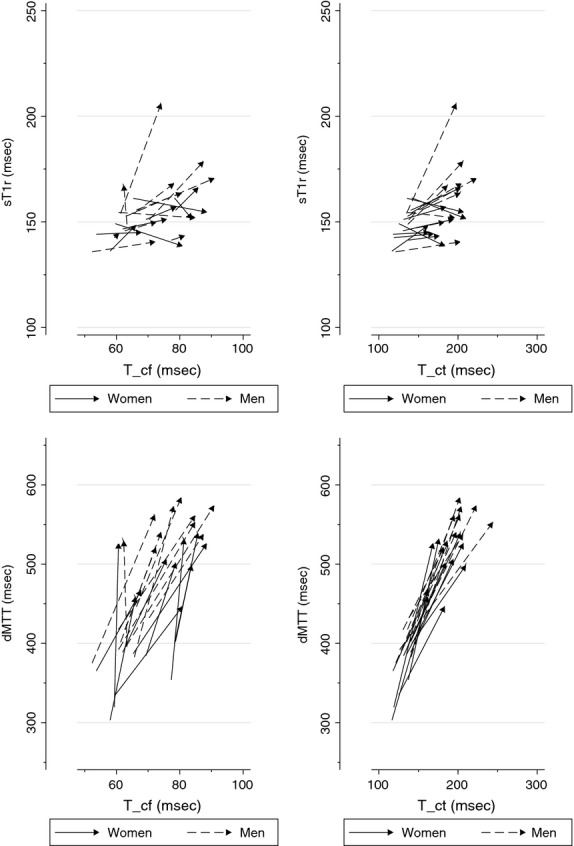
Timing indices derived from the aortic pulse plotted as a function of carotid-to-femoral and carotid-to-distal propagation times in individual subjects studied in two body positions. sT1r time to onset of systolic reflected wave, dMTT mean transit time of diastolic reflected wave, *T*_cf_ carotid-to-femoral propagation time, *T*_ct_ carotid-to-tibial propagation time. Data points from the same subject joined by an arrow, with origin corresponding to Head-up and arrowhead to Head-down position. sT1r and dMTT means of two determinations made immediately before and immediately after the measurements of *T*_cf_ and *T*_ct_.

In Figure5, the forward propagation times *T*_cf_ and *T*_ct_ measured in each subject and each position are plotted against the corresponding timing indices independently obtained from the analysis of the central aortic pulse waveform. It may be seen that, on changes in body position, these forward propagation times covary with both sT1r and dMTT. This relationship is especially tight between *T*_ct_ and dMTT. We used mixed model linear regression to evaluate the slopes of the relationships between either sT1r or dMTT, treated as dependent variables, and *T*_cf_ or *T*_ct_ (predictors). As shown in Table5, all estimated slopes (*β*) were significantly different from zero (*P* ≤ 0.001) and positive. Interestingly, the regression of dMTT on *T*_ct_ yielded a slope close to 2, indicating that each change (increment or decrement) in the carotid-to-tibial propagation time translated to twice that change in the timing of the diastolic wave seen on the aortic pressure pulse.

**Table 5:** Relationships between the timing indices derived from the analysis of the central aortic pulse waveform and actual propagation delays.

	Predictor	
	*T*_cf_	*T*_ct_
Dependent variable		
sT1r		
*β*	0.48	0.14
CI	0.20–0.76	0.07–0.22
*P*	<0.001	<0.001
dMTT		
*β*	5.8	2.2
CI	4.6–6.9	1.9–2.3
*P*	<0.001	<0.001

Results obtained with mixed model linear regression, using the data shown in Figure5. sT1r time to return of the reflection wave of the aortic waveform, dMTT diastolic mean transit time, *T*_cf_ carotid-to-femoral propagation delay, *T*_ct_ carotid-to-tibial propagation delay. *β* regression coefficients, indicating the estimated increase in the dependent variable for each unit increase in the predictor. CI 95% confidence interval for *β*.

### Differences related to gender

Statistically significant gender-related differences in these basic hemodynamic data are displayed in the upper part of Table4. In women compared to men, peripheral but not central systolic BP was lower in both positions, and the switch from Head-down to Head-up induced a more marked rise in peripheral or central diastolic BP. in accordance with the expected lower orthostatic tolerance of women (Convertino 1998; Cheng et al. 2011).

PWV did not differ, in either position, between men and women (Fig.4, Table4), in accordance with the recent literature (Reference Values for Arterial Stiffness Collaboration 2010; Zhang et al. 2013). In contrast, sAix was significantly larger in women (Fig.3, Table4), again according to expectation (Mitchell et al. 2004; McEniery et al. 2005; Heim et al. 2013). The femoral-to-tibial propagation times as well as the transit times of the reflected waves obtained from the aortic pulse were larger in men (Figs.3, 4, Table4), reflecting their larger body size (Table1).

## Discussion

In the present study, the timing indices of reflected pressure waves in the central aorta – as derived from radial artery tonometry – were compared with the forward propagation times of the arterial pressure pulse toward the lower body half – as directly measured with an independent method. The major new finding is that the former and the latter are closely correlated, at least in young subjects in the course of an orthostatic stress test. This result supports the validity of interpreting morphologic features of the central aortic pressure waveform in terms of superimposed forward and reflected waves, even when approximately reconstructed from the noninvasive recording of the radial pulse.

Prior to our study, very little information was available regarding the impact of body position on PWV and transmission times of pressure waves toward the lower limb. An ancient study by Kroeker et al. compared the timing of radial and femoral pulse in the supine ant 70° Head-up position, but suffers from methodological shortcomings (Kroeker and Wood 1955). More recently, Huijben et al. (2012) reported that tilting healthy volunteers from supine to 60° Head-up caused a 15% increase of PWV_cf_. To our knowledge, the only prior work having investigated the effect of orthostatic stress on PWV in arteries of the lower limb is that by Hasegawa and Rodbard (1979). These authors examined volunteers at different tilt angles, in each position obtaining simultaneous recordings of pressure pulses in the brachial and posterior tibial artery. The values of PWV thus deduced, and presumably influenced by transmission velocities in lower limb arteries, increased massively from −30° Head-down tilt (9.9 m/sec) to the fully erect position (17.6 m/sec). Our study is the first to have simultaneously evaluated the impact of orthostatic stress on arterial PWV in both the thoracoabdominal (i.e. PWV_cf_) and lower limb compartments (PWV_ft_). The present results are in striking accord with these previous data, in both direction and magnitude. The angular change from Head-down to Head-up was roughly the same as in the study by Huijben et al. and caused a similar moderate increase in PWV_cf_ (by 14% in women and 20% in men, Fig.4). By contrast, the impact on PWV_ct_ and especially PWV_ft_ was quite marked, although not as massive as in the observations by Hasegawa and Rodbard who tested their subjects with a more extreme change of body position.

Transmural pressure (Ptm, the difference between intraluminal and extramural pressure) is a major determinant of PWV in fluid-filled tubes with walls of nonlinear stress–strain relationship such as the arteries (Nichols et al. 2011b). Thus, the first-line explanation for the observed impact of body position on local PWV is likely to reside in the induced modifications of Ptm. Available data in humans indicate that, in the course of orthostatic stress, the variation in intraluminal pressure at any site in the arterial tree is entirely determined by the hydrostatic factor, that is, it equals the change in the vertical height of the fluid column between this site and the heart (multiplied by the volumic mass of blood) (Katkov and Chestukhin 1980; Meyer et al. 2002; Rosales-Velderrain et al. 2011; Gemignani et al. 2012). In contrast, extramural pressure follows the hydrostatic factor only very incompletely, at least in the lower limb (Pfeffer et al. 2001; Meyer et al. 2002). These considerations support that, on switching from Head-down to Head-up, Ptm increased in arteries of the lower body half, and plausibly more so in leg than in abdominal and pelvic vessels, thus explaining both the direction and the relative magnitude of the observed changes in PWV_cf_ and PWV_ft_.

Whether corrected for heart rate or not, and irrespective of gender, the sAix was greater (i.e., less negative) in the Head-down than in the Head-up position (Fig.3A). In their work cited above, Huijben et al. (2012) have made a similar finding, namely that in healthy elderly volunteers examined supine and at increasing degrees of Head-up tilt, the sAix@75 was highest in the supine position. The dependence of sAix on body position has also been studied without the use of a tilt table. Two independent groups have tested the impact of passive leg elevation in supine subjects. One reported that this maneuver consistently increased the sAix@75 (Heffernan et al. 2010) while the other made the opposite finding (Kamran et al. 2009). Finally, in two different protocols from our laboratory, this index was lower in supine than sitting young healthy subjects similar to those of the present study (Jaccoud et al. 2012; Heim et al. 2013). Taken together, these data show that changes in body position with the common denominator of increasing the vertical height of the legs with respect to the trunk and head (“legs-up” vs. “legs-down”) do not have uniform effects on systolic augmentation of the aortic pulse. The reasons for these discrepancies are unclear.

Especially in young healthy humans, the diastolic profile of the central arterial pulse often departs markedly from the monoexponential decay predicted by the simple windkessel model (Kelly et al. 1989; Reesink et al. 2007; Nichols et al. 2011c; Heim et al. 2013), and data from the present study are no exception in that respect (Fig.2). This feature has commonly been ascribed to the occurrence of reflected waves, reaching the aorta after termination of ventricular ejection, possibly from the lower body half (Nichols et al. 2011c; Segers et al. 2012), but other interpretations are a priori possible. First, since it starts in the immediate proximity of aortic valve closure (although not coinciding with it, discussed in Heim et al. 2013), the diastolic wave could be caused by the convective deceleration of ejected blood and concomitant transformation of kinetic into potential energy according to the Bernouilli principle. It has been posited that this factor alone cannot account for the observed amplitude of the upward convexity of the central pulse in diastole (Wang and Parker 2004), but this argument is based on a computer simulation without experimental verification. A second source of uncertainty on the nature of the diastolic waves arises when the central waveforms are inferred from the radial pulse, as carried out here and in our previous studies (Jaccoud et al. 2012; Heim et al. 2013). It has been pointed out that the generalized transfer function used to that effect is only an approximation (Gallagher et al. 2004), and in such conditions a calculation artifact is hard to exclude.

In the face of these theoretical objections, the present study provides additional support to the interpretation of diastolic waves as due to reflections, even when observed on central waveforms reconstructed from the radial pulse, and that such reflections originate in the lower body half. First, concomitant with the longer forward propagation times toward the lower limb, the dMTT was markedly higher in the Head-down, compared to the Head-up position (Fig.3B). Analogously, we have previously found, in young subjects, a higher dMTT in the supine, compared to the sitting position (Jaccoud et al. 2012; Heim et al. 2013). Furthermore, Reesink et al. have used M-mode echography of the carotid artery to evaluate, among other parameters, the timing of the diastolic distension wave (a surrogate for the pressure wave) in this vessel, which is closer to the central aorta than is the radial artery. Consistent with our results, they reported that the diastolic wave recorded in this fashion was also delayed in young subjects when examined supine, compared to sitting (Reesink et al. 2007). Collectively, these observations indicate a coherent effect of various body postures on the timing of diastolic waves recorded with different methods, such that these are delayed in “legs-up,” compared to “legs-down” positions.

The present study is unique in allowing direct comparisons to be made between the timing of the diastolic wave (i.e., dMTT) and the independently measured forward propagation times of pulse waves toward the lower limb. Strikingly, the changes induced by tilting in dMTT and *T*_cd_ were closely coupled, as graphically shown in Figure3, with the former being about twice the latter within a narrow confidence interval (Table5). With the caveat that they represent no more than an association, these results nevertheless strongly support that the diastolic waves observed on central aortic pulses reconstructed from radial tonometric recordings indeed correspond to pressure waves reflected from the lower body half.

### Differences related to gender

Because we expected that the hemodynamic response to tilt would not be the same in women and men (Huxley 2007), we stratified the study according to gender. Indeed, the shift from Head-down to Head-up induced less increase in diastolic blood pressure and less decrease in stroke volume in women, compared to men, although the observed change in heart rate was similar in both groups (Tables2–4), a pattern in similar to observations by Schroeder et al. (Schroeder et al. 2004). However, regarding aortic waveform and PWV parameters, the only statistically discernible interactions of position with gender were for sT1r and possibly *T*_ct_ (Table4), while the size of the corresponding effects was minimal. These data suggest that, in terms of arterial wave propagation and augmentation of aortic pressure by reflected waves, the responses to tilting do not greatly differ between men and women, at least in this young healthy population.

### Study limitations

We have used the generalized transfer function (GTF) approach in order to reconstruct the central pulse from recordings of the radial pulse. This approach has not been specifically validated during tilting. However, the aortic pressure reconstructed from the radial pulse by means of the GTF has been found to agree reasonably well with its directly measured counterpart in a wide range of physiological conditions, including Valsalva maneuver, abdominal compression, vena cava obstruction, and vasodilator infusion (Chen 1997; Florian et al. 2013). Having said that, authors have mentioned that the constancy of the transfer function across subjects and conditions is an approximation whose accuracy is best for the low-frequency components of the pressure signal, such that the reproduction of features containing high frequencies, such as the systolic augmentation wave (and therefore the calculated aortic sAix), might be less accurate (Segers et al. 2000; Laurent 2007). This constitutes a clear limitation to our study. However, the postural changes in the transit times of reflected waves (derived from the calculated aortic waveform) correlated reasonably well with changes in the forward propagation times measured with a different method (Fig.5, Table5), arguing for the validity of the GTF in our experimental conditions.

Another limitation of the present study relates to the measurements of forward propagation times and PWV, because the pulses in the carotid artery and in the aorta travel in opposite directions. Thus, when the systolic upstroke is sensed on the carotid site, the forward wave has already traveled some distance from the aortic arch toward the lower half of the body. In other words, as measured in the study, *T*_cf_ and *T*_ct_ underestimate the true propagation times from the aortic arch to the femoral (*T*_aa_f) and the tibial artery (*T*_aa_t), by an amount equal to the propagation time from the aortic arch to the carotid site of measurement (*T*_aa_c). This fact is usually taken into account in the calculation of PWV, by subtracting the sternal notch to carotid from the sternal notch to femoral or tibial recording sites (Laurent et al. 2006). We have not used this correction, which may have biased our results for PWV_cf_ toward high values (Weber et al. 2009). However, the error on travel distance should not affect the comparisons made between the two body positions in the same subject. A more subtle issue arises when considering *T*_cf_ and *T*_ct_ as surrogates for *T*_aa_f and *T*_aa_t, respectively, as we have done. As stated above, *T*_aa_f and *T*_aa_t are thereby underestimated by an amount equal to *T*_aa_c. Again, if this error remained constant across conditions in the same subject, our conclusions regarding the impact of tilting on these travel times would remain unaffected. At first sight, a gravitational influence on *T*_aa_c it possible, with a scenario running as follows: on shifting from Head-up to Head-down, hydrostatic forces would lead to an increase in carotid artery transmural pressure, thus to a higher local PWV, thus to a shorter *T*_aa_c. In such conditions, the lengthening of *T*_cf_ and *T*_ct_ observed in subjects placed Head-down, compared to Head-up, might be driven in part by a modification of *T*_aa_c. However, this effect is likely to be too small to affect our conclusions, because tilting affects the vertical height of the carotid measurement site to a much lesser extent, compared to the vertical height of the femoral and tibial measurement sites.

Finally, body position had a large influence on stroke volume (Tables2 and 3), presumably therefore on the pattern of ventricular ejection. How this factor has influenced the recorded aortic pressure waveforms in uncertain, because we have not carried out any recording of instantaneous aortic flow.

In summary, we have provided substantive evidence that, even when observed on a reconstructed version of the central aortic pulse (i.e., calculated from a tonometric recording of the radial pulse), deviations from a monoexponential decay pattern during diastole are due to reflected pressure waves, and therefore are not an artifact of the reconstruction algorithm. With tilting, the transit times of these waves are modified in a manner closely coupled to concomitant changes in forward propagation times toward the lower limbs, suggesting a significant contribution of the latter as reflection sites.

## Figures and Tables

**Figure 6:**
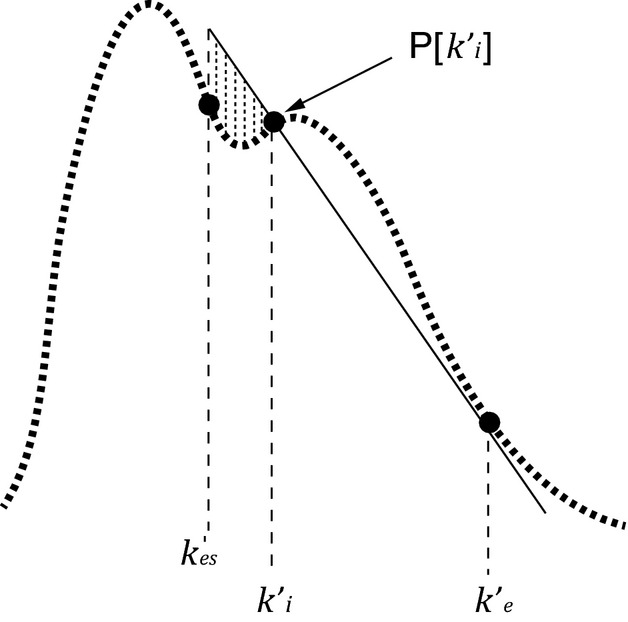
Landmarks for iterative evaluation of onset and end of diastolic wave.

## Glossary

dAix: *Diastolic augmentation index*. An estimate of the amplitude of the reflected wave seen on the diastolic part of the aortic pressure profile. Expressed in percent of pulse pressure. Calculated as described in Figure1, by custom software, from exported waveforms previously recorded by the SphygmoCor device.
dMTT: *Mean transit time of the diastolic reflected wave*. An index of the timing of the diastolic reflected wave. Calculated as described in Figure1, by custom software, from exported waveforms previously recorded by the SphygmoCor device.
ED: *Ejection time*. Duration of ventricular ejection. Calculated by the SphygmoCor software from the central aortic pulse waveform.
sAix: *Systolic augmentation index*. An estimate of the amplitude of the reflected wave seen on the systolic part of the aortic pressure profile. Expressed in percent of pulse pressure. Calculated by the SphygmoCor software from the central aortic pulse waveform.
sAix@75: sAix corrected for the influence of heart rate. Calculated by the SphygmoCor software.
sT1r: Time from the initial systolic upstroke of aortic pressure to the onset of the reflected wave reaching the aorta in systole. Calculated by the SphygmoCor software from the central aortic pulse waveform.
*T*_cf_: *Carotid-to-femoral forward propagation time*. Time lag between the simultaneously recorded carotid and femoral arterial pulses. Calculated by the Complior device.
*T*_ct_: *Carotid-to-tibial forward propagation time*. Time lag between the simultaneously recorded carotid and tibial arterial pulses. Calculated by the Complior device.
*T*_cf_: *Carotid-to-femoral forward propagation time*. Time lag between the simultaneously recorded femoral and tibial arterial pulses. Calculated by the Complior device.
ti: Time from the initial systolic upstroke of aortic pressure to the onset of the reflected wave reaching the aorta in diastole. Calculated by custom software (see Appendix).
te: Time from the initial systolic upstroke of aortic pressure to the end of the reflected wave reaching the aorta in diastole. Calculated by custom software (see Appendix).

## Appendix

### Algorithm to delineate the diastolic wave

The algorithm takes as input: (1) the array P containing *n* successive points of the aortic pressure pulse sampled at a frequency of 128 Hz (represented by the dotted waveform in the figure), and (2) the index in this array of the last systolic point (*kes*), as provided in the export from the SphygmoCor software. The output consists of the indexes *ki* and *ke*, which, respectively, designate to the first and last point of the diastolic wave.

The algorithm starts with a first estimate *k′i* of *ki*, and iterates to its final output, as follows:

1. Set *k′i* = *kes + (n – kes)/10*
2. Among all straight lines passing through the point P[*k′i*] and intersecting the diastolic profile to the right of this point, find the one with the most negative slope. If, as usual, the last part of the profile has an upward concavity, it will be touched by this line in a tangent fashion at P[*k′e*]. If not, set *k′e = n*
3. If, inbetween *kes* and *k′i*, the line (any point of it) is above the pressure profile (stippled area in Fig.A1), then:

Set *k′i = k′i – 1;*
Go to step 2
Else
Set *ki* = *k′i;*
Set *ke* = *k′e;*
Stop

The software displays the aortic waveform and the final line, for approval by the operator, based on the criterion that the line should be tangent to the pressure profile at P[*ki*], and also at P[*ke*] if the diastolic profile has a final upward concavity. If the algorithm appears to have failed, the line can be displaced interactively, with recalculation of *ki* and *ke*. This manual correction was required in 11 of the 240 recordings analyzed in the present study.

## References

[b1] Chen CH (1997). Estimation of central aortic pressure waveform by mathematical transformation of radial tonometry pressure. Validation of generalized transfer function. Circulation.

[b2] Cheng YC, Vyas A, Hymen E, Perlmuter LC (2011). Gender differences in orthostatic hypotension. Am. J. Med. Sci.

[b3] Convertino VA (1998). Gender differences in autonomic functions associated with blood pressure regulation. Am. J. Physiol. Regul. Integr. Comp. Physiol.

[b4] Delachaux A, Waeber B, Liaudet L, Hohlfeld P, Feihl F (2006). Profound impact of uncomplicated pregnancy on diastolic, but not systolic pulse contour of aortic pressure. J. Hypertens.

[b5] Dischl B, Engelberger RP, Gojanovic B, Liaudet L, Gremion G, Waeber B (2011). Enhanced diastolic reflections on arterial pressure pulse during exercise recovery. Scand. J. Med. Sci. Sports.

[b6] Dow P, Hamilton WF (1939). An experimental study of the standing waves in the pulse propagated through the aorta. Am. J. Physiol.

[b7] Florian JP, Simmons EE, Chon KH, Faes L, Shykoff BE (2013). Cardiovascular and autonomic responses to physiological stressors before and after six hours of water immersion. J. Appl. Physiol.

[b8] Frank O (1899). Die Grundform des Arteriellen Pulses (translated by Sagawa K, Lie RK, Schaefer J, 1990, J Mol Cell Cardiol 22:253–277). Z. Biol.

[b9] Frank O (1905). Der Puls in den Arterien. Z. Biol.

[b10] Gallagher D, Adji A, O'Rourke MF (2004). Validation of the transfer function technique for generating central from peripheral upper limb pressure waveform. Am. J. Hypertens.

[b11] Gemignani T, Matos-Souza JR, Franchini KG, Nadruz W (2012). Leg blood pressure measured in orthostatic posture is associated with left ventricular mass in normotensive subjects. Am. J. Hypertens.

[b12] Gojanovic B, Waeber B, Gremion G, Liaudet L, Feihl F (2009). Bilateral symmetry of radial pulse in high-level tennis players: implications for the validity of central aortic pulse wave analysis. J. Hypertens.

[b13] Hasegawa M, Rodbard S (1979). Effect of posture on arterial pressures, timing of the arterial sounds and pulse wave velocities in the extremities. Cardiology.

[b14] Heffernan KS, Sharman JE, Yoon ES, Kim EJ, Jung SJ, Jae SY (2010). Effect of increased preload on the synthesized aortic blood pressure waveform. J. Appl. Physiol.

[b15] Heim A, Liaudet L, Waeber B, Feihl F (2013). Pulse wave analysis of aortic pressure: diastole should also be considered. J. Hypertens.

[b16] Huijben AM, Mattace-Raso FU, Deinum J, Lenders J, van den Meiracker AH (2012). Aortic augmentation index and pulse wave velocity in response to head-up tilting: effect of autonomic failure. J. Hypertens.

[b17] Huxley VH (2007). Sex and the cardiovascular system: the intriguing tale of how women and men regulate cardiovascular function differently. Adv. Physiol. Educ.

[b18] Jaccoud L, Rotaru C, Heim A, Liaudet L, Waeber B, Hohlfeld P (2012). Major impact of body position on arterial stiffness indices derived from radial applanation tonometry in pregnant and non pregnant women. J. Hypertens.

[b19] Kamran H, Salciccioli L, Gusenburg J, Kazmi H, Ko EH, Qureshi G (2009). The effects of passive leg raising on arterial wave reflection in healthy adults. Blood Press. Monit.

[b20] Katkov VE, Chestukhin VV (1980). Blood pressure and oxygenation in different cardiovascular compartments of a normal man during postural exposures. Aviat. Space Environ. Med.

[b21] Kelly R, Hayward C, Avolio A, O'Rourke M (1989). Noninvasive determination of age-related changes in the human arterial pulse. Circulation.

[b22] Kroeker EJ, Wood EH (1955). Comparison of simultaneously recorded central and peripheral arterial pressure pulses during rest, exercise and tilted position in man. Circ. Res.

[b23] Laurent S (2007). How to estimate central pressure augmentation?. J. Hypertens.

[b24] Laurent S, Cockcroft J, Van Bortel L, Boutouyrie P, Giannattasio C, Hayoz D (2006). Expert consensus document on arterial stiffness: methodological issues and clinical applications. Eur. Heart J.

[b25] Mader U, Martin BW, Schutz Y, Marti B (2006). Validity of four short physical activity questionnaires in middle-aged persons. Med. Sci. Sports Exerc.

[b26] McDonald DM (1955). The relation of pulsatile pressure to flow in arteries. J. Physiol.

[b27] McEniery CM, Yasmin, Hall IR, Qasem A, Wilkinson IB, Cockcroft JR (2005). Normal vascular aging: differential effects on wave reflection and aortic pulse wave velocity: the Anglo-Cardiff Collaborative Trial (ACCT). J. Am. Coll. Cardiol.

[b28] Meyer RS, White KK, Smith JM, Groppo ER, Mubarak SJ, Hargens AR (2002). Intramuscular and blood pressures in legs positioned in the hemilithotomy position: clarification of risk factors for well-leg acute compartment syndrome. J. Bone Joint Surg. Am.

[b29] Mitchell GF, Parise H, Benjamin EJ, Larson MG, Keyes MJ, Vita JA (2004). Changes in arterial stiffness and wave reflection with advancing age in healthy men and women: the Framingham Heart Study. Hypertension.

[b30] Nichols WW, Conti CR, Walker WE, Milnor WR (1977). Input impedance of the systemic circulation in man. Circ. Res.

[b31] Nichols WW, O'Rourke MF, Vlachopoulos C, Nichols WW, O'Rourke MF, Vlachopoulos C (2011a). Chap. 1: Introduction. McDonald's blood flow in arteries.

[b32] Nichols WW, O'Rourke MF, Vlachopoulos C, Nichols WW, O'Rourke MF, Vlachopoulos C (2011b). Chap. 3: Properties of the arterial wall: theory. McDonald's blood flow in arteries.

[b33] Nichols WW, O'Rourke MF, Vlachopoulos C, Nichols WW, O'Rourke MF, Vlachopoulos C (2011c). Chap. 9: Wave reflections. McDonald's blood flow in arteries.

[b34] Nichols WW, O'Rourke MF, Vlachopoulos C, Nichols WW, O'Rourke MF, Vlachopoulos C (2011d). Chap. 10: Contours of pressure and flow waves in arteries. McDonald's blood flow in arteries.

[b35] O'Rourke MF (1967). Pressure and flow waves in systemic arteries and the anatomical design of the arterial system. J. Appl. Physiol.

[b36] O'Rourke MF, Hashimoto J (2007). Mechanical factors in arterial aging: a clinical perspective. J. Am. Coll. Cardiol.

[b37] O'Rourke MF, Yaginuma T (1984). Wave reflections and the arterial pulse. Arch. Intern. Med.

[b38] Parker KH (2009). A brief history of arterial wave mechanics. Med. Biol. Eng. Comput.

[b39] Pfeffer SD, Halliwill JR, Warner MA (2001). Effects of lithotomy position and external compression on lower leg muscle compartment pressure. Anesthesiology.

[b40] Reesink KD, Hermeling E, Hoeberigs MC, Reneman RS, Hoeks AP (2007). Carotid artery pulse wave time characteristics to quantify ventriculoarterial responses to orthostatic challenge. J. Appl. Physiol. Respir. Environ. Exerc. Physiol.

[b41] Reference Values for Arterial Stiffness Collaboration (2010). Determinants of pulse wave velocity in healthy people and in the presence of cardiovascular risk factors: ‘establishing normal and reference values’. Eur. Heart J.

[b42] Rosales-Velderrain A, Cardno M, Mateus J, Kumar R, Schlabs T, Hargens AR (2011). Toe blood pressure and leg muscle oxygenation with body posture. Aviat. Space Environ. Med.

[b43] Schroeder C, Adams F, Boschmann M, Tank J, Haertter S, Diedrich A (2004). Phenotypical evidence for a gender difference in cardiac norepinephrine transporter function. Am. J. Physiol. Regul. Integr. Comp. Physiol.

[b44] Segers P, Carlier S, Pasquet A, Rabben SI, Hellevik LR, Remme E (2000). Individualizing the aorto-radial pressure transfer function: feasibility of a model-based approach. Am. J. Physiol. Heart Circ. Physiol.

[b45] Segers P, Swillens A, Vermeersch S (2012). Reservations on the reservoir. J. Hypertens.

[b46] Taylor MG (1957). An approach to an analysis of the arterial pulse wave II. Fluid oscillations in an elastic pipe. Phys. Med. Biol.

[b47] Wang JJ, Parker KH (2004). Wave propagation in a model of the arterial circulation. J. Biomech.

[b48] Weber T, Ammer M, Rammer M, Adji A, O'Rourke MF, Wassertheurer S (2009). Noninvasive determination of carotid-femoral pulse wave velocity depends critically on assessment of travel distance: a comparison with invasive measurement. J. Hypertens.

[b49] Wesseling KH, Jansen JR, Settels JJ, Schreuder JJ (1993). Computation of aortic flow from pressure in humans using a nonlinear, three-element model. J. Appl. Physiol.

[b50] Westerhof N, Lankhaar JW, Westerhof BE (2009). The arterial Windkessel. Med. Biol. Eng. Comput.

[b51] Wormersley JR (1958). Oscillatory flow in arteries II: the reflection of the pulse wave at at junctions and rigid inserts in the arterial system. Phys. Med. Biol.

[b52] Zhang Y, Agnoletti D, Protogerou AD, Topouchian J, Wang JG, Xu Y (2013). Characteristics of pulse wave velocity in elastic and muscular arteries: a mismatch beyond age. J. Hypertens.

